# Takayasu’s Arteritis in Pregnancy: Challenges During the Ongoing Coronavirus Disease 2019 Pandemic for Optimal Maternal and Neonatal Outcomes

**DOI:** 10.7759/cureus.12386

**Published:** 2020-12-30

**Authors:** Sweta Singh, Aditya Pati, Sudipta Mohakud, Dibya Ranjan Behera

**Affiliations:** 1 Obstetrics and Gynecology, All India Institute of Medical Sciences, Bhubaneswar, IND; 2 Radiology, All India Institute of Medical Sciences, Bhubaneswar, IND; 3 Cardiology, All India Institute of Medical Sciences, Bhubaneswar, IND

**Keywords:** takayasu’s arteritis, ct angiography, covid-19

## Abstract

Takayasu’s arteritis is a rare systemic vasculitis affecting the aorta and its primary branches. In developing countries, it is often associated with late diagnosis and high morbidity and mortality. We report a case of Takayasu’s arteritis diagnosed in the postpartum period and discuss the challenges faced for optimal outcomes during the ongoing coronavirus disease 2019 pandemic.

## Introduction

Takayasu’s arteritis is a rare systemic vasculitis affecting the aorta and its primary branches [1]. The annual incidence is 0.4 to 2 per million population per year, with a female preponderance [2,3]. Often, there are significant delays in diagnosis and management. We report a case of Takayasu’s arteritis in pregnancy diagnosed in the postpartum period and discuss the challenges faced for optimal outcomes during the ongoing coronavirus disease 2019 (COVID-19) pandemic.

## Case presentation

A 21-year-old unbooked multigravida at 30 weeks of gestation was referred with preeclampsia and anhydramnios. Her first pregnancy had resulted in a spontaneous miscarriage at 14 weeks. Her second pregnancy was complicated with preeclampsia and intrauterine fetal death at 28 weeks of gestation. During the current pregnancy, she was on tablet labetalol 100 mg thrice daily and low-dose aspirin for chronic hypertension. Earlier, she had been treated with antihypertensive agents telmisartan and amlodipine during the inter-pregnancy period.

**Figure 1:**
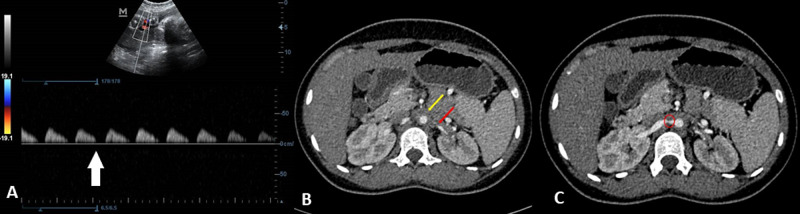
(A) Absent end-diastolic flow (white arrow) in the fetal umbilical artery on color Doppler ultrasound. (B) CECT arterial-phase image (axial section) showing narrowing of the maternal abdominal aortic lumen with hypodense wall thickening (yellow arrow) and severe narrowing of the left renal artery (red arrow). (C) CECT arterial-phase image of the maternal abdomen (axial section) showing severe focal stenosis of the proximal right renal artery (red circle). CECT, contrast-enhanced computed tomography

At presentation, her pulse rate was 96 beats per minute and blood pressure was 146/92 mmHg in the right arm in the supine position. There was no pallor or edema. Cardiovascular and respiratory system examinations were unremarkable. Uterus corresponded to 28 weeks of gestation, and the fetal heart rate was 142 beats per minute. Obstetric ultrasound revealed a single live intrauterine fetus in cephalic presentation with estimated fetal weight less than the third centile, nil liquor, and absent end-diastolic flow on umbilical artery Doppler, suggestive of fetal growth restriction stage II (Figure 1A).

Her hemoglobin was 12.3 g/dL and serum creatinine was 0.7 mg/dL. Erythrocyte sedimentation rate and other acute-phase reactants were not determined.

She was admitted and treated with four doses of dexamethasone 6 mg intramuscular 12 hours apart for fetal lung maturity and magnesium sulfate 4 g intravenous loading dose followed by 1 g each hour for 24 hours for fetal neuroprotection. The dose of tablet labetalol was increased to 200 mg thrice daily. However, due to uncontrolled hypertension, she underwent an emergency caesarean section after 48 hours of admission. A preterm live male baby weighing 1,300 g and good Apgar score was delivered. The baby had a neonatal intensive care stay of 10 days. Intraoperatively, her blood pressure was 167/110 mmHg in the left arm and 154/95 mmHg in the right arm. In view of chronic hypertension and intraoperative differential blood pressure in both the arms, Takayasu’s arteritis was considered, and the patient underwent further workup. No bruit was audible. Proteinuria was 1+, and the urine albumin:creatinine ratio was 2.0. Fundoscopy revealed grade 1 hypertensive retinopathy.

**Figure 2:**
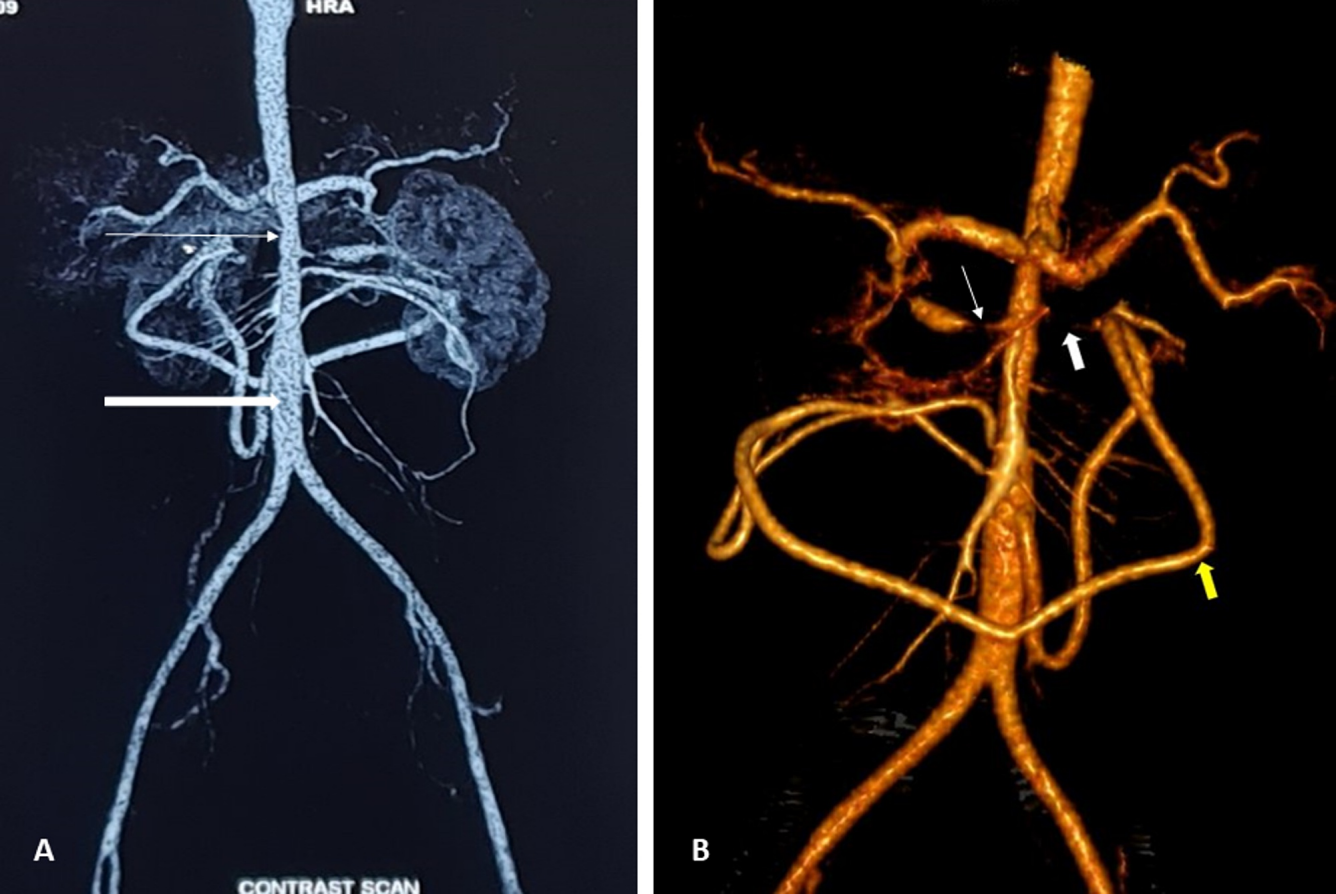
CECT arterial-phase image. (A) Significant luminal narrowing of the distal descending thoracic aorta (thin white arrow) with poststenotic dilatation (thick white arrow). (B) Three-dimensional volume-rendered image showing grossly stenosed right renal artery (thin white arrow) with poststenotic dilatation. The proximal left renal artery is not visualized (thick white arrow) due to severe narrowing. The inferior mesenteric artery is hypertrophied (yellow arrow) and anastomosing with the branches of the superior mesenteric artery. CECT, contrast-enhanced computed tomography

CT angiography of bilateral renal vessels revealed concentric circumferential wall thickening of the distal descending thoracic aorta (Figure 1B) and bilateral proximal renal artery stenosis (Figure 1C). The descending thoracic aorta was stenosed over a length of 13 cm with significant luminal narrowing (Figure 2A), as well as the presence of poststenotic dilatation in the aorta and the right renal artery (Figure 2B).

Her final diagnosis was angiographic type III Takayasu’s arteritis, with inactive disease determined using Kerr’s score. She was planned for a stenting procedure in the postpartum period. However, due to the lockdown and closure of routine services in view of the COVID-19 pandemic, the proposed procedure has been deferred. She is currently on antihypertensives, is lactating, and is under follow-up by teleconsultation. Both the mother and the baby are doing well.

## Discussion

Takayasu’s arteritis, a rare systemic large vessel vasculitis, is associated with high morbidity and mortality [1]. While it is a disease commonly seen in young women, actual incidence during pregnancy is quite rare [3]. Hence, treating obstetricians may not be readily familiar with the diagnostic criteria, clinical activity scoring, and management during pregnancy. Diagnosis is often delayed in developing countries due to the insidious onset of the disease and nonspecific early symptoms [2]. Furthermore, the COVID-19 pandemic has complicated matters due to lockdown and disruptions in routine services.

Takayasu’s arteritis is diagnosed using the American College of Rheumatology criteria, wherein the presence of three or more of the following criteria is diagnostic: (a) age at onset less than or equal to 40 years, (b) claudication of an extremity, (c) decreased brachial artery pulse, (d) difference in systolic blood pressure between arms greater than 10 mmHg, (e) a bruit over the subclavian arteries or the aorta, and (f) arteriographic evidence of narrowing or occlusion of the entire aorta, its primary branches, or large arteries in the proximal upper or lower extremities [4]. In developing countries, postpartum renal artery Doppler ultrasound of women with hypertension during pregnancy helps in diagnosing new cases. Increasingly, imaging is thought to be the major determinant of disease activity in Takayasu’s arteritis [5]. Furthermore, the ongoing pandemic, with disruption in routine care, complicated matters, and the patient presented to us late.

Angiographically, the disease presents as the following five types: type I, branches from the aortic arch; type II, ascending aorta, aortic arch, and its branches (IIa), and thoracic descending aorta (IIb); type III, thoracic descending aorta, abdominal aorta, and/or renal arteries; type IV, abdominal aorta and/or renal arteries; type V, combined features of type IIb and IV are involved [6]. Our patient had angiographic type III disease; however, during pregnancy, type V disease is most commonly described [3]. The disease is marked by periods of remission and active disease. Active disease, as determined using Kerr’s criteria, is the presence of more than two of the following criteria: (a) constitutional symptoms, (b) new bruits, and (c) increased acute-phase reactants or new angiographic features [7]. Alternately, the Indian Takayasu clinical activity score is also used [8]. During pregnancy, majority of the women are in remission, and pregnancy does not interfere with disease progression [3].

During pregnancy, Takayasu’s arteritis has the potential to cause severe maternal and neonatal complications such as chronic hypertension, superimposed preeclampsia, preterm labor, preterm induction of labor, spontaneous miscarriage, fetal growth restriction, increased caesarean delivery, and placental abruption [3,9,10]. However, recently, with prepregnancy diagnosis, tight preconceptional disease control, strict follow-up, targeted treatment of hypertension, and multidisciplinary approach, positive pregnancy outcomes are increasingly being reported, with no cardiovascular adverse events [2,3,9,10]. However, this may be affected during the ongoing COVID-19 pandemic.

Treatment in pregnancy is with antihypertensives, glucocorticoids to control the inflammation, low-dose aspirin, and fetomaternal consultations for the assessment of fetal growth restriction.

## Conclusions

We presented the case of a 21-year-old multipara with type III Takayasu’s arteritis diagnosed in the postpartum period. While the obstetric outcome was good, early diagnosis helps in tailoring optimal management. The ongoing COVID-19 pandemic has complicated matters as multidisciplinary approach, referrals, and treatment got disrupted.

## References

[1] Pacheco RL, Latorraca COC, de Souza AWS, Pachito DV, Riera R (2017). Clinical interventions for Takayasu arteritis: a systematic review. Int J Clin Pract.

[2] Gudbrandsson B, Wallenius M, Garen T, Henriksen T, Molberg Ø, Palm Ø (2017). Takayasu arteritis and pregnancy: A population-based study on outcomes and mother/child-related concerns. Arthritis Care Res (Hoboken).

[3] David LS, Beck MM, Kumar M, Rajan SJ, Danda D, Vijayaselvi R (2020). Obstetric and perinatal outcomes in pregnant women with Takayasu’s arteritis: single centre experience over five years. J Turk Ger Gynecol Assoc.

[4] Arend WP, Michel BA, Bloch DA (1990). The American College of Rheumatology 1990 criteria for the classification of Takayasu arteritis. Arthritis Rheum.

[5] Kenar G, Karaman S, Çetin P (2020). Imaging is the major determinant in the assessment of disease activity in Takayasu’s arteritis. Clin Exp Rheumatol.

[6] Moriwaki R, Noda M, Yajima M, Sharma BK, Numano F (1997). Clinical manifestations of Takayasu arteritis in India and Japan--new classification of angiographic findings. Angiology.

[7] Kerr GS, Hallahan CW, Giordano J, Leavitt RY, Fauci AS, Hoffman GS (1994). Takayasu arteritis. Ann Intern Med.

[8] Misra R, Danda D, Rajappa SM (2013). Development and initial validation of the Indian Takayasu Clinical Activity Score (ITAS2010). Rheumatology (Oxford).

[9] Kirshenbaum M, Simchen MJ (2018). Pregnancy outcome in patients with Takayasu’s arteritis: cohort study and review of the literature. J Matern Fetal Neonatal Med.

[10] Tanaka H, Tanaka K, Kamiya C, Iwanaga N, Yoshimatsu J (2014). Analysis of pregnancies in women with Takayasu arteritis: complication of Takayasu arteritis involving obstetric or cardiovascular events. J Obstet Gynaecol Res.

